# Paleoenvironments of Late Devonian tetrapods in China

**DOI:** 10.1038/s41598-023-47728-y

**Published:** 2023-11-21

**Authors:** Xuelian Guo, Gregory J. Retallack, Jinhao Liu

**Affiliations:** 1 Key Laboratory of Western China’s Mineral Resources of Gansu Province, School of Earth Sciences, Lanzhou University, Lanzhou, 730000 People’s Republic of China; 2 Department of Earth Sciences, University of Oregon, Eugene, OR 97403-1272 USA

**Keywords:** Ecology, Palaeoecology, Climate sciences, Palaeoclimate, Coevolution, Palaeontology

## Abstract

The major evolutionary transition from fish to amphibian included Late Devonian tetrapods that were neither fish nor amphibian. They had thick necks and small limbs with many digits on elongate flexuous bodies more suitable for water than land. Habitats of Devonian tetrapods are of interest in assessing selective pressures on their later evolution for land within three proposed habitats: 1, tidal flats, 2, desert ponds, and 3, woodland streams. Here we assess paleoenvironments of the Late Devonian tetrapod *Sinostega* from paleosols in Shixiagou Canyon near Zhongning, Ningxia, China. Fossil tetrapods, fish, molluscs, and plants of the Zhongning Formation are associated with different kinds of paleosols, representing early successional vegetation, seasonal wetlands, desert shrublands, and riparian woodlands, and paleoclimates ranging from semiarid moderately seasonal to monsoonal subhumid. The tetrapod *Sinostega* was found in a paleochannel of a meandering stream below a deep-calcic paleosol supporting well drained progymnosperm woodland in a monsoonal subhumid paleoclimate. This habitat is similar to that of the tetrapods *Densignathus*, *Hynerpeton*, and an indeterminate watcheeriid from Pennsylvania, USA. Chinese and Pennsylvanian Late Devonian tetrapods lived in productive woodland streams, choked with woody debris as a refuge from large predators. Habitats of other Devonian tetrapods have yet to be assessed from studies of associated paleosols as evidence for their ancient climate and vegetation.

## Introduction

Although incompleteness of the fossil record of early vertebrate colonization of the land is lamentable^1^, persistent attention to rare fossils and their sedimentary context have revealed intermediate forms within other major evolutionary transitions such as dinosaur to bird^2^ and ape to human^3^. The late Jenny Clack proposed an extended phase of Late Devonian tetrapods intermediate within the evolutionary transition from fish to amphibian^4^. Basal tetrapods *Ichthyostega* and *Acanthostega* from Greenland were medium sized (0.5–1 m long) with long sinuous bodies, lateral line canals, and short limbs inadequate for rapid locomotion on land^1,4^. High-carriage, fully terrestrial, tetrapods evolved much later during the Early Carboniferous^4^. Late Devonian was also a culmination of Middle Devonian evolution of progymnosperm and cladoxyl forests^5^, so that short multidirectional limbs of basal tetrapods may have been exaptations for navigating and hiding in woody debris of streams, rather than adaptations to walking or hauling on land^6^. Alternatively these stumpy limbs have been regarded as adaptations for hauling out of shrinking desert ponds^7^, or on the slimy mud of tidal flats^8^.

**Figure 1:**
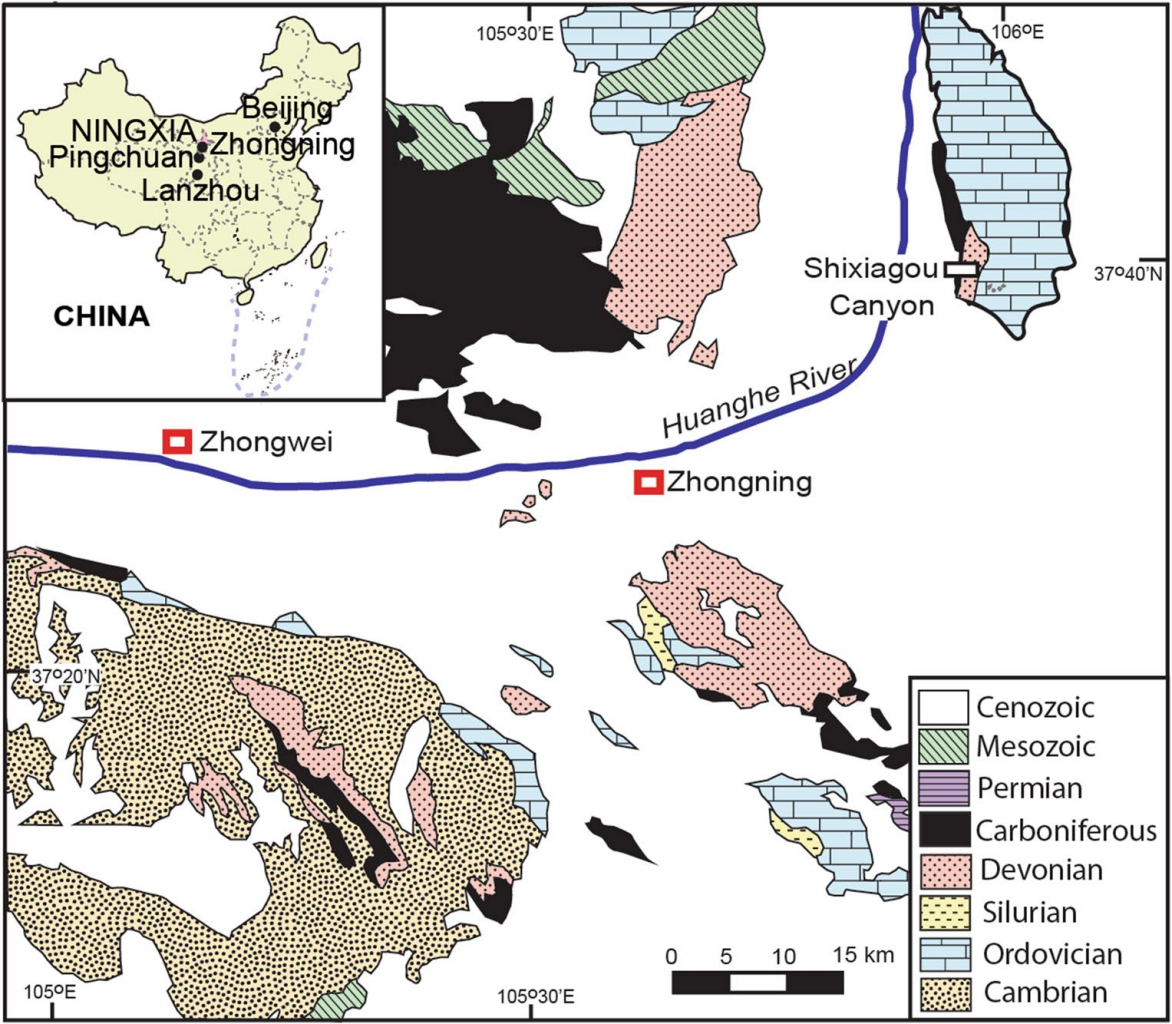
Late Devonian tetrapod locality and Shixiagou Canyon section near Zhongning, Ningxia Hui Autonomous Region, China. Geological map has been simplified^34^.

**Figure 2:**
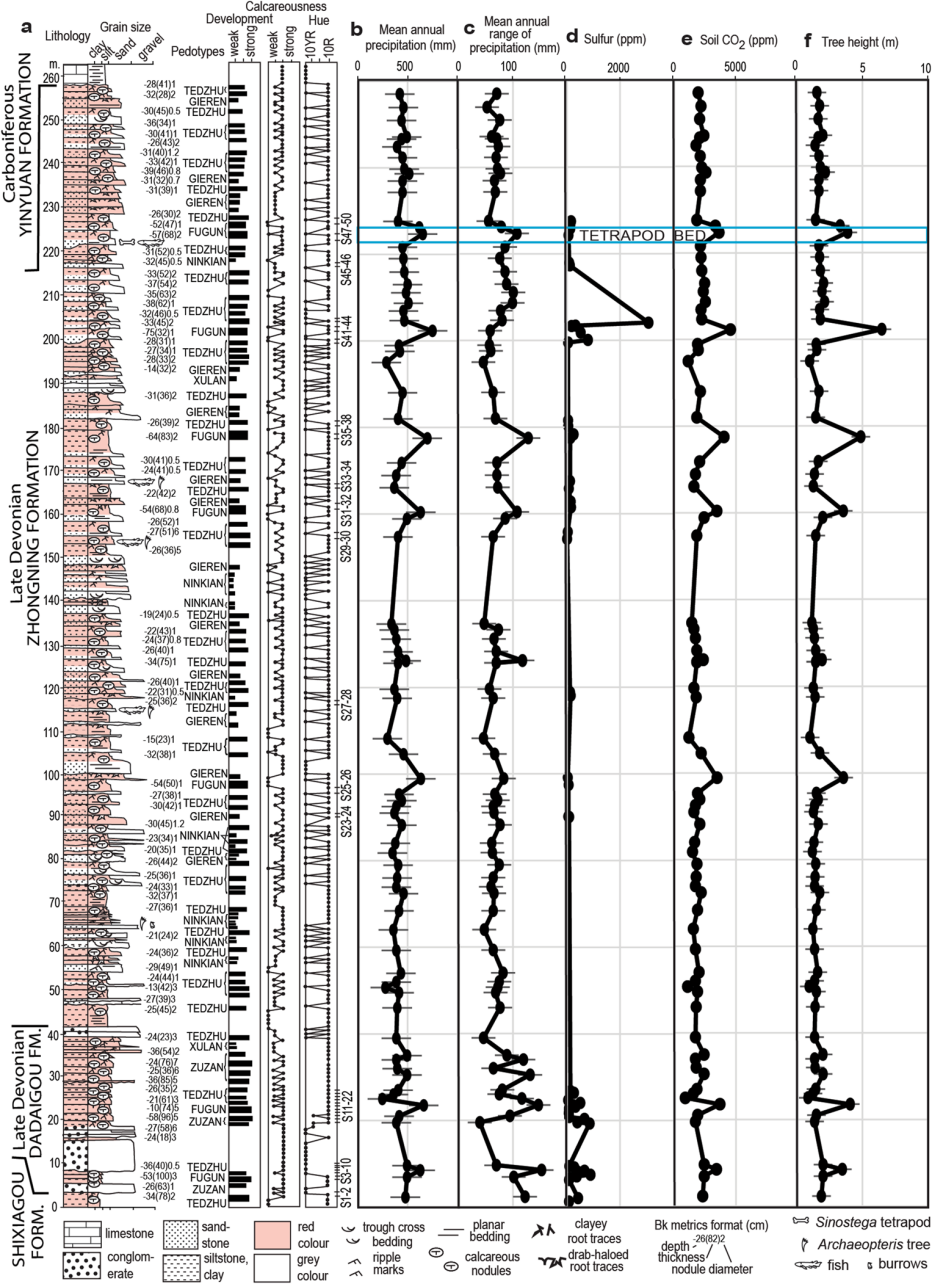
Geological section and paleoclimatic proxies for the Late Devonian Zhongning Formation of China: (**a**) measured section noting levels and degrees of development of paleosols, reaction with HCl and Munsell hue; (**b**) Mean annual precpitation (mm) estimated from compaction-corrected depth to calcic horizon^30,32^ (**c**) Mean annual range of precipitation (mm) from compaction-corrected thickness of calcic horizon^30,32^ (**d**) sulfur (ppm) analysed by XRF; (**e**) Soil CO_2_ (ppm) and (**f**) tree height (m), both estimated from compaction-corrected depth to calcic horizon^5,31^. Data sources are supplementary Information Tables S1–S4).

This paper evaluates these alternative scenarios from evidence of Late Devonian paleosols at a tetrapod locality in China (Fig. 1). The Chinese Late Devonian tetrapod *Sinostega*^9^ is based on an almost complete jaw from the Zhongning Formation in Shixiagou Canyon, northeast of Zhongning County, Ningxia Hui Autonomous Region (222 m in Fig. 2). The size of a complete *Sinostega* animal can be estimated at a little more than 0.63 ± 0.1 m from the size of its jaw relative to other early tetrapods known from complete skeletons^6^. *Sinostega* was thus similar in size to the Famennian Greenland tetrapod *Acanthostega*, which it most resembles^9^. *Sinostega* was part of an evolutionary radiation of Devonian tetrapodomorphs^10–23^, and about the same geological age as *Ichthyostega*, *Acanthostega*, *Densignathus*, *Hynerpeton*, and *Metaxygnathus* (Table 1). Like these other tetrapods, *Sinostega* may have had larger pelvis, sacrum and digits than Frasnian *Tiktaalik*^20^, but lacked the short body and high carriage of *Tulerpeton*^10^ and Carboniferous amphibians^4^.

**Table 1:** Devonian tetrapodomorph skeletal remains in temporal order.

Age	Ma	Taxon	Locality	References
Famennian	360	*Tulerpeton curtum*	Andreyevka, Russia	^10^
	363	*Acanthostega gunnari*, *Ichthyostega* sp. indet	Gauss Halvø, Greenland	^11^
	366	*Ichthyostega stensioei*	Gauss Halvø, Greenland	^11^
	366	*Densignathus rowei, Hynerpeton basseti*, Whatcheeriid	Hyner, Pennsylvania	^12^
	366	*Metaxygnathus denticulatus*	Bunduburrah, New South Wales	^13^
	366	*Sinostega pani, Hongyu chowi*	Shixiagou, China	^9,14^
	366	cf. *Ichthyostega* indet	Strud, Belgium	^11^
	366	*Ventastega curonica*	Ketleri, Latvia	^11^
	366	*Ventastega curonica*	Pavāri, Latvia	^11^
	370	*Jacubsonia livnensis*	Gornostayevka, Russia	^15^
	370	*Tutusius umlambo, Umzantsia amazana*	Waterloo Farm, South Africa	^16^
	372	Tetrapoda indet	Plucki, Poland	^17^
	372	*Parmastega aelidae*	Sosnogorzsk, Russia	^18^
Frasnian	373	*Obruchevichthys gracilis*	Novgorod, Russia	^19^
	373	*Obruchevichthys gracilis*	Velna-Ala, Latvia	^19^
	375	*Elginerpeton pancheni*	Scat Craig, Scotland	^19^
	377	*Tiktaalik roseae*	Blind Fiord, Nunavut	^20^
	379	*Gogonasus andrewsae*	Gogo, Western Australia	^21^
	379	*Panderichthys rhombolepis, Livoniana multidentata*	Lode, Latvia	^22^
Givetian	380	*Elpisostege watsoni*	Miguasha, Quebec	^23^

**Figure 3:**
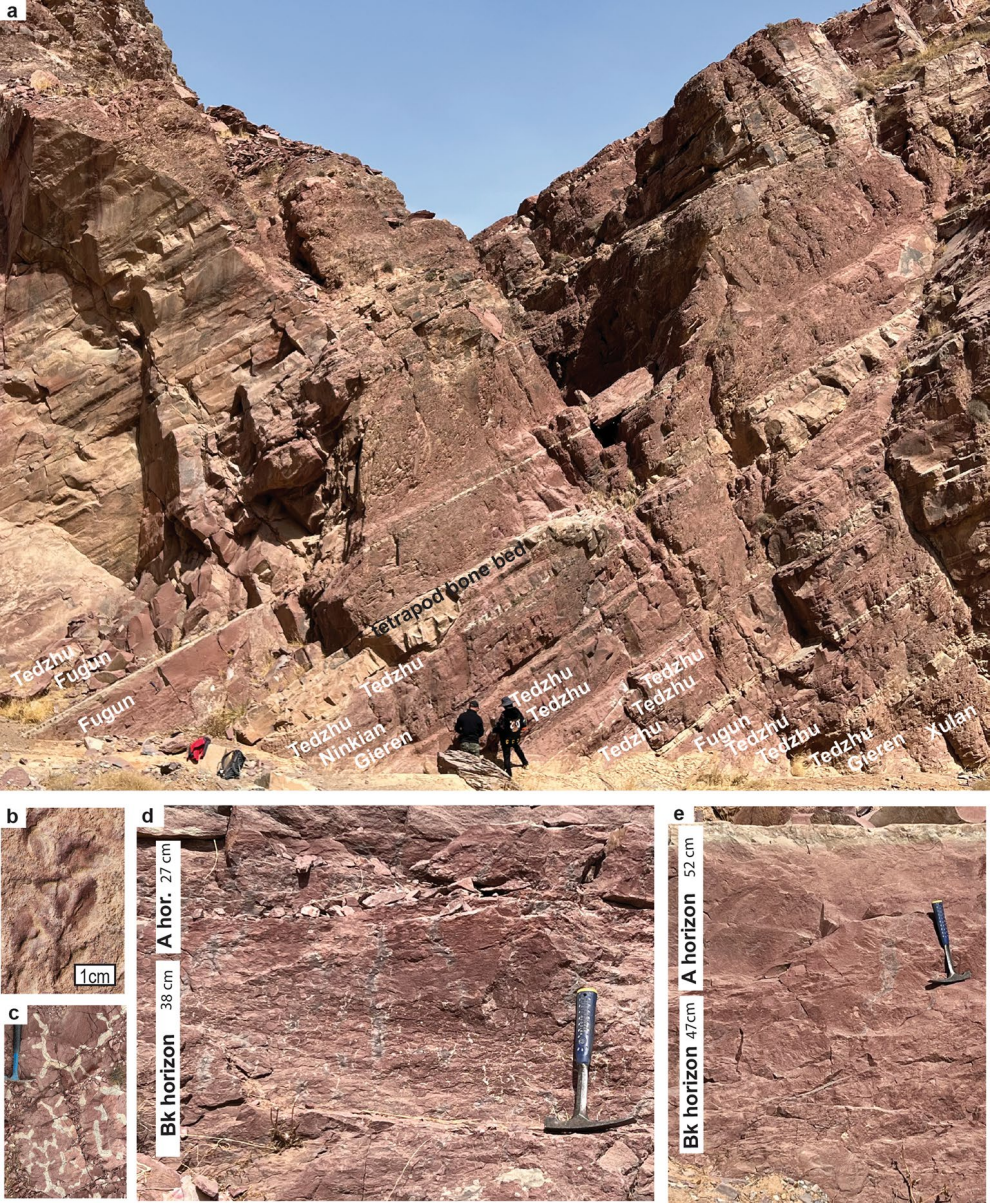
Field photos of Late Devonian paleosols and root traces in the Zhongning Formation of China; (**a**) sandstone paleochannel yielding the tetrapod *Sinostega pani*^9^, and paleosols above and below in Shixiagou Canyon; (**b**) progynosperm short shoot of *Archaeopteris macilenta* (63 m in Fig. 2); (**c**) drab-haloed dichotomizing rhizome system (− 10 m below Fig. 2); (**d**) shallow-calcic paleosol of Tedzhu pedotype (91 m in Fig. 2); (**e**) deep-calcic paleosol of Fugun pedotype (222 m in Fig. 2).

Other fossils from the Zhongning Formation (Supplementary Information Table S1), include the Givetian to Frasnian progymnosperm tree *Archaeopteris macilenta*, represented by rare leafy twigs (Fig. 3b) as well as abundant woody root traces (Fig. 3d–e). We consider *A. macilenta* a senior synonym of “*Sphenopteris taihuensis*”, previously recorded from the Zhongning and Shixiagou Formations^24^. Also notable is *Ningxiaphyllum trilobatum*^24^, lobed cupules with three dense ovoid bodies comparable with the late Famennian early seed fern *Archaeosperma arnoldi* from Pennsylvania^25^. Sinolepid fishes in the Zhongning Formation also are evidence of late Famennian age^26^. Oxygen and sulfur stable isotopic composition of Famennian sarcopterygian and placoderm fish from Shixiagou reveal that they lived in estuaries of mixed salinity, or returned to streams from the sea to spawn, but that study did not analyse the tetrapod *Sinostega*^27^.

## Results

### Ancient soilscapes

Both the Zhongning and Shixiagou Formation in Ningxia and the Shaliushui Formation near Pingchuan in Gansu^28^ are long sequences of superimposed red calcareous paleosols (Fig. 3), similar to sequences of Late Devonian paleosols of Pennsylvania and New York^5,6^. Interbedded with the paleosols are sandstone palaeochannels, such as the tetrapod bed itself, with cut banks as deep as 2 m (Fig. 3a). The deepest parts of the incised tetrapod fluvial palaeochannel return at a wavelength of 11.5 m laterally along outcrop (Fig. 3a), as evidence of a sinuous meandering stream, like those found elsewhere in Middle to Late Devonian red beds^5^.

The main effort of our study was to document and then interpret the variety of paleosols in a long geological section (Fig. 2), measured in Shixiagou Canyon (from narrows at N37.654774° E105.9983626° to tetrapod locality at N37.654735° E105.994513°). Six distinct kinds of paleosol encountered were given descriptive pedotype names using the indigenous Dongxiang language^29^, and their description and interpretation is detailed in supplementary information online Tables S2 and S3. In summary, some pedotypes are very weakly developed, shaley (Ninkian) or sandy (Xulan), or weakly developed (Gieren), with much lamination, ripple marks and other sedimentary features persisting, despite partial disruption by root traces. Their root traces are mostly deeply reaching as evidence of good drainage, but one Ninkian profile had dichotomizing drab-haloed rhizomes confined to one horizon as if waterlogged (Fig. 3c). These profiles represent early successional vegetation of habitats disturbed by regular flooding such as levees and point bars^5,12^. Other profiles are characterized by pedogenic carbonate nodules that are shallow (< 40 cm) within profiles on conglomerate (Zuzan) or siltstones (Tedzhu: Fig. 3d), or deep (> 40 cm) within the profiles below a clayey siltstone horizon (Fugun, Fig. 3e). These three calcareous pedotypes have abundant, stout, deeply-reaching root traces (Fig. 3d–e) as evidence for woody vegetation of progymnosperms or pteridosperms in well established communities stable for the many thousands of years needed to create such large nodules^30^. Most of the calcareous paleosols have shallow calcic horizons, but 7 stratigraphic levels have paleosols with deep-calcic horizons (Fig. 2). The bed with the tetrapod *Sinostega pani* is in the uppermost of these exceptional, deep-calcic, paleosol horizons.

### Ancient vegetation

Paleoclimate and vegetation of deep-calcic paleosols can be quantified by a variety of measurements of the paleosols (Fig. 2b–c,e–f), such as calcic horizon proxies for vegetation stature^5^ and productivity^31^, and chemical composition as proxies for mean annual precipitation and temperature^32^. Modern soils with deeper calcic horizons have taller trees^5^ and higher soil respiration of CO_2_ as an index of secondary productivity^31^. The relationship of Devonian progymnosperm tree height to soil carbonate depth differs from modern seed plants^5^, and for estimates of past height needs correction for burial estimated from local stratigraphic thickness and vitrinite reflectance of coal in overlying beds^33–36^. By this metric (see Methods), progymnosperms of the two deep calcic (Fugun) paleosols near the tetrapod level were 6.5 ± 0.7 m and 3.9 ± 0.7 m tall. This was a woodland rather than forest in some classifications^37^, in strong contrast to desert shrubland heights of 0.9–2.2 m for the many shallow calcic (Tedzhu and Zuzan) paleosols in the same sequence (Figs. 2, 3a).

This indication of woodlands around streams at the Chinese tetrapod locality can be supplemented with estimates from paleosols of secondary soil productivity^31^ as respired CO_2_ from compaction-corrected depth to calcic horizon. By this proxy, communities of the two deep calcic (Fugun) paleosols near the tetrapod level had high CO_2_ levels of 7228 ± 588 ppm and 9101 ± 588 ppm, whereas desert shrublands of the many shallow calcic (Tedzhu and Zuzan) paleosols had only 1763 ± 588 ppm to 5128 ± 588 ppm (Fig. 2e). Woodlands with tetrapods and taller trees were more productive than usual during accumulation of the rest of the Zhongning Formation.

### Ancient climate

Part of the explanation for transiently increased productivity was increased mean annual precipitation and mean annual temperature, which can be estimated from chemical index of alteration without K_2_O and alkali index (see Methods) from chemical analysis of non-calcareous parts of paleosols^32^. Unfortunately, only two of the analysed specimens have CaO low enough to apply these proxies (Supplementary Information Table S5), but they give subhumid (692 ± 181 and 858 ± 181 mm) precipitation and temperate (5.0 ± 4.4 and 4.8 ± 4.4 °C) temperatures for paleosols at 19.6 and 20.8 m (in Fig. 2). This is cool for an estimated tropical palaeolatitude (0.9 ± 8.8°) for the Zhongning Formation^38^, and this location may have been at high elevation during Late Devonian mountain-building to the south^34^.

Supporting indications of subhumid precipitation and of high mountains nearby come from measurements of carbonate nodules corrected for compaction. Depth to carbonate nodules is also related to mean annual precipitation (MAP)^30^, and thickness of carbonate nodular horizon is related to mean annual range of precipitation (MARP), or difference between wettest and driest month. The two deep-calcic (Fugun) paleosols near the tetrapod horizon have subhumid MAP of 651 ± 147 and 749 ± 147 mm, and moderately monsoonal MARP of 108 ± 22 mm and 100 ± 22 mm, whereas the numerous shallow-calcic (Tedzhu and Zuzan) paleosols have semiarid MAP (246 ± 147–516 ± 147 mm) and non-monsoonal MARP (39 ± 22–94 ± 22 mm).

## Discussion

Transients of deep calcic paleosols at irregular intervals within long sequences of shallow calcic paleosols have been found in other parts of the world, such as Devonian of Australia^39^ and New York-Pennsylvania^5,6^, Permian–Triassic of South Africa^40^ and Utah^41^, and Cretaceous of China^42^ and Nevada^41^. In all of these cases, deep calcic levels are correlative with global CO_2_ greenhouse spikes, and named marine black shales, such as 366 Ma annulata event^8^, which may correlate with the Shixiagou tetrapod site. They are considered to represent massive atmospheric pollution by CO_2_ and CH_4_ from basaltic eruptions of Large Igneous Provinces. In the case of late Famennian, large basaltic eruptions of the Dniepr-Donetz Rift in Ukraine may be to blame^43^. A volcanic component to the deep calcic spikes at the tetrapod locality near Zhongning is indicated by anomalous enrichment of sulfur (Fig. 2d), mercury and chlorine^44^ (Supplementary Information Tables S5–7). While massive eruption may explain the rapid onset of greenhouse spikes, their abatement requires exceptional carbon burial by geographic expansion of more productive warm-wet communities and soils polewards and into deserts^45^. Woodlands with tetrapods presumably existed in more humid regions upslope and to the west throughout deposition of the Zhongning Formation, but only during these greenhouse spikes did they expand geographically into the area currently in Shixiagou Canyon.

Comparable studies of paleosols associated with other basal tetrapods remain to be completed, but existing evidence is compatible with the view that they were largely aquatic creatures of meandering streams in productive semiarid to subhumid woodlands, venturing onto land only during floods and the wet season^6,46^. Desert shrubland paleosols also were found in the Devonian^5^, but evidence presented here and elsewhere^6,8^, suggest that basal tetrapods avoided them. There is also isotopic, sedimentary, and paleontological evidence of Devonian tetrapods in intertidal to estuarine settings^1,11,16,27^. Middle Devonian (Eifelian, ca 392 Ma) trackways from Zachelmie in Poland have been used to argue for intertidal tetrapod habitats^8^, but these lack digits and gait of genuine tracks, and are more like fish feeding traces^47^. Zachelmie paleoenvironment has been reinterpreted as lakes and floodplain, rather than intertidal^48,49^. Similar doubts have been voiced about Middle Devonian (Givetian, ca 382 Ma) Valentia Island trackways of Ireland^50^, which have body drag marks, and are similar to terrestrial locomotion trails of lungfish^51,52^.

**Figure 4:**
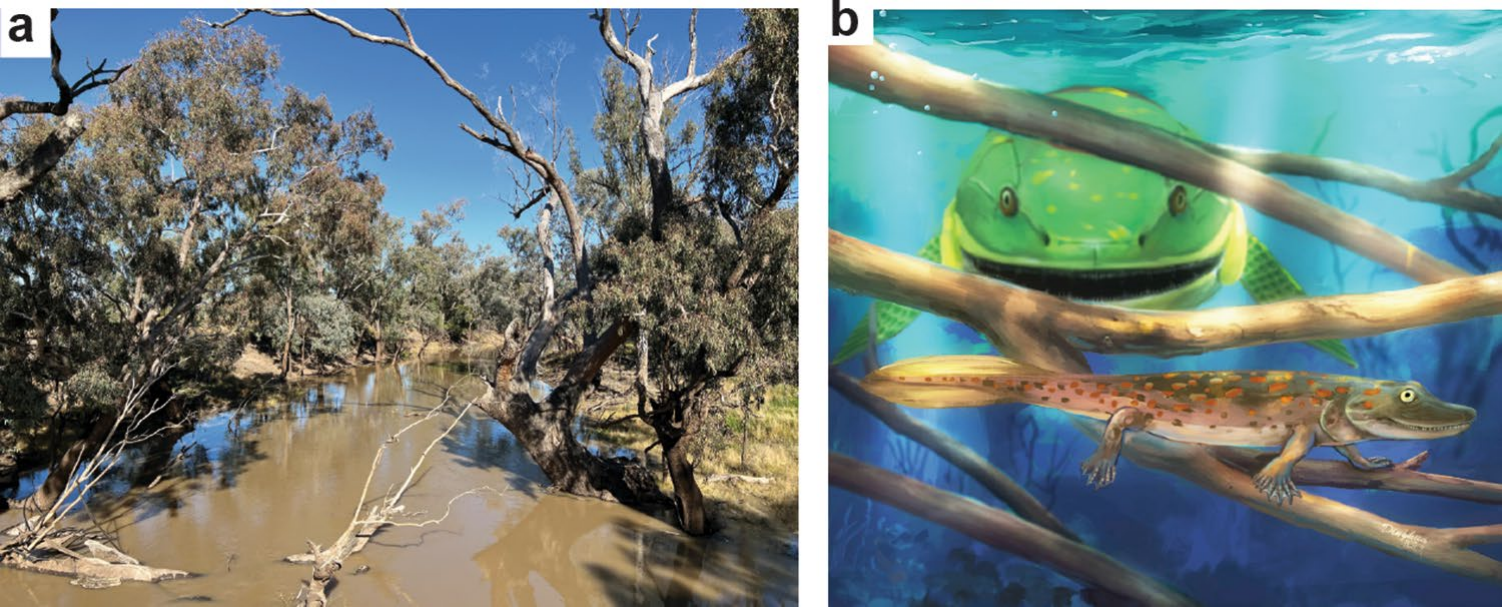
Analogous modern environment (**a**) and reconstruction of *Sinostega pani* (**b**); (**a**) Turragulla Creek, 4 km north of Pilliga, New South Wales, Australia. Pilliga box (*Eucalyptus pillagensis*) forms temperate dry woodland (MAP 571 mm, MAT 17.6 °C), and woody debris in the creek (S30.3049529° E148.8203385°), as an analogous modern environment for Chinese^9^ and Pennsylvanian^12^ Late Devonian tetrapods. (**b**) reconstruction of *Sinostega pani*^9^ hiding from large *Hongyu chowi*^14^, behind woody debris. Reconstruction by Dinghua Yang.

The woodland hypothesis^6^ links Late Devonian tetrapod evolution to Middle to Late Devonian evolution of forests^5,46,53^. Newly evolved forests changed fluvial hydrology for fish and amphibians, because trees stabilized banks to create meandering, perennial, deep streams, rather than braided, ephemeral, shallow streams^5,46^. Paleosols and palaeobotany of the tetrapod bed near Zhongning are remarkably similar to those of tetrapod localities in Pennsylvania^6,8^, also in streams flanked and littered with dry woodland debris, like modern creeks in outback New South Wales, Australia (Fig. 4a). Woody debris in and around streams improved fish and amphibian diversity and abundance with alternating slow and fast-flowing sections, and cooler and deeper pools^54–56^. Tetrapods could have navigated woody debris with their small limbs in order to avoid predation from much larger fish such as *Hongyu*^14^ (Fig. 4b) and *Hyneria*^12^ found in the same deposits. In modern streams and lakes, salamanders take refuge from predation by fish in woody debris^57,58^. The importance of woody debris for modern amphibians is now increasingly appreciated from studies of human deforestation^57,58^. Woody debris may have been critical to early amphibian evolution.

## Methods

The main activity of this project was measuring a detailed section of paleosols by the method of eyeheights adjusted by cosine from dip of 30^o^W on strike azimuth184^o^ (Fig. 2). Three key features were recorded: (1) destruction of sedimentary bedding and size of pedogenic carbonate nodules as proxies for soil development, (2) reaction with 0.1 M HCl as a proxy carbonate content, and (3) Munsell hue as a record of chemical oxidation. Depth and thickness of the calcic horizons were also measured in the field (Bk metrics of Fig. 2 and Supplementary Information Table S4). No experiments with animals were part of this research. All data generated are included within this article.

Six distinct kinds of paleosols, or pedotypes, were recognized in the field (Supplementary Information Tables S2–3). Major and trace element concentrations were measured using XRF spectroscopy. Each XRF measurement was on a compacted disk, derived from whole-rock powder sieved to 200 μm mesh and weighing at 4 g, on a VP-320 XRF spectrometer. Mercury (Hg) was analysed by atomic fluorescence spectrometry. The relative standard deviation is about 1% (Supplementary Information Tables S4–7).

The relationship between jaw length (*J* in mm) and body length (*L* in m) of complete skeletons of early tetrapods^6^ was calculated using Eq. 1 (n = 6, r^2^ = 0.98, s.e. =  ± 0.05, *p* = 0.006).

1 $$ L = 0.0056J - 0.017 $$

Progymnosperm tree height was calculated from Eq. 2 (n = 9, r^2^ = 0.95, s.e. =  ± 0.7, *p* = 0.00002). Depth to carbonate (*D* in cm) in this equation needs to be corrected for compaction^33^ (*C* in %) due to burial (*B* in km) using Eq. 3 based on 1 km up-section to Ningxia coal^34^ with vitrinite reflectance of 0.74%^35^, so suffered about 3 km burial depth^36^.

2 $$ H = 0.6351e^{0.0182D} $$

3 $$ C = \frac{ - 0.62}{{\left\{ {\left( {\frac{0.38}{{e^{{\frac{B}{0.17}}} }}} \right) - 1} \right\}}} $$

Paleosols can be used to infer secondary soil productivity as respired CO_2_ (*S* in ppm) estimated from compaction-corrected (Eq. 3) depth to calcic horizon (*D* in cm) using Eq. 4 (n = 17, r^2^ = 0.54, s.e. =  ± 588, *p* = 0.00002) from modern soils^31^.

4 $$ S = 35.3D + 588 $$

Mean annual precipitation (*MAP* in mm) and mean annual temperature (*MAT* in °C) can be estimated for paleosols from chemical index of alteration without K_2_O (*K* as 100Al_2_O_3_/[Al_2_O_3_ + CaO + Na_2_O] in moles) and alkali index (*A*, as molar K_2_O + Na_2_O/Al_2_O_3_) of non-calcareous parts of paleosols^32^ by Eqs. 5 (r^2^ = 0.72, s.e. =  ± 182 mm, *p* = 0.00001) and 6 (r^2^ = 0.37, s.e. =  ± 4.4 °C, *p* = 0.00001).

5 $$ MAP = 221.1e^{0.0197C} $$

6 $$ MAT = - 18.5S + 17.3 $$

Mean annual precipitation in calcareous soils of unconsolidated loess and alluvium of plains, can be inferred from depth to carbonate nodules (D in cm)^30^ (MAP in mm) by Eq. 7 (r^2^ = 0.52, s.e =  ± 147 mm, *p* = 0.00001). Mean annual range of precipitation (MARP as mm difference between wettest and driest month mean) is related to the thickness of the Bk horizon (H in cm) of soils in unconsolidated sediment of plains, again with robust statistics (r^2^ = 0.58, s.e. =  ± 22 mm, *p* = 0.00001), by Eq. 8.

7 $$ MAP = 137.24 + 6.45D - 0.0132D^{2} $$

8 $$ MARP = 0.79H + 13.7 $$

### Supplementary Information

Supplementary Tables.

## Data Availability

All data generated or analyzed during the current study are included in this published article and its supplementary information files, but GR can provide additional information on request (gregr@uoregon.edu).
