# Supplementary material for: Paleoenvironments of Late Devonian tetrapods in China
Source: Sci Rep. 2023 Nov 21;13:20378. doi: 10.1038/s41598-023-47728-y (PMC10663569; doi:10.1038/s41598-023-47728-y)
Supplement: Supplementary file 1 — Supplementary Tables. [file 41598_2023_47728_MOESM1_ESM.docx]

Supplementary information for “Paleoenvironments of Late Devonian tetrapods in China”

by Xuelian Guo, Gregory J. Retallack and Jinhao Liu

**Table S1.** Fossils of the Late Devonian Zhongning Formation

| Latin name | Common name or affinities | Level (m) | Ref. |
| --- | --- | --- | --- |
| *Calamospora atava* | Rhyniopsid, Barinophyte, Lycopsid, Equisetopsid, or Noeggerathian spores | 250 | 1,2 |
| *Calamospora nigrata* | Rhyniopsid, Barinophyte, Lycopsid, Equisetopsid, or Noeggerathian spores | 250 | 1,2 |
| *Punctatisporites* | As above, but plausibly also Pteridosperm prepollen | 250 | 1,2 |
| *Retusotriletes distinctus* | Rhyniopsid or Zosterophyll spore | 250 | 1,2 |
| *Apiculatisporis microconus* | Zygopterid fern spore | 250 | 1,2 |
| *Granulatisporites minusculus* | Rhyniopsid, Botryopterid or Dipterid fern spore | 250 | 1,2 |
| *Acanthotriletes uncatus* | Uncertain pteridophyte spore | 250 | 1 |
| *Apiculiretusispora plicata* | Bryopsid, Rhyniopsid, or Zosterophyll spore | 250 | 1,2 |
| *Apiculiretusispora nitida* | Bryopsid, Rhyniopsid, or Zosterophyll spore | 250 | 1,2 |
| *Apiculiretusispora septalata* | Bryopsid, Rhyniopsid, or Zosterophyll spore | 250 | 1,2 |
| *Verrucosisporites omalus* | Lycopsid or Filicopsid spore, or Pteridosperm prepollen | 250 | 1,2 |
| *Verruciretusispora robusta* | Uncertain pteridophyte spore | 250 | 1 |
| *Verruciretusispora magnifica* | Uncertain pteridophyte spore | 250 | 1 |
| *Chelinospora* | Bryopsid or Rhyniopsid spore | 250 | 1, 2 |
| *Stenozonotriletes extensus* | Uncertain pteridophyte spore | 250 | 1 |
| *Stenozonotriletes laevigatus* | Uncertain pteridophyte spore | 250 | 1 |
| *Stenozonotriletes conformis* | Uncertain pteridophyte spore | 250 | 1 |
| *Cymbosporites truncatus* | Lycopsid spore | 250 | 1 |
| *Aneurospora greggsii* | Rhyniopsid, Barinophyte, Protolepidodendrale or Progymnosperm spore | 250 | 1,2 |
| *Geminospora devonicus* | Archaeopterid progymnosperm spore | 250 | 1,2 |
| *Geminospora lemurata* | Archaeopterid progymnosperm spore | 250 | 1,2 |
| *Archaeozonotriletes agnatus* | Lycopsid or Archaeopterid progymnosperm spore | 250 | 1,2 |
| *Archaeozonotriletes variabilis* | Lycopsid or Archaeopterid progymnosperm spore | 250 | 1,2 |
| *Ancyrospora furcula* | Trimerophtye or Progymnosperm spore | 250 | 1,2 |
| *Hymenozonotriletes brevimammus* | Uncertain pteridophyte spore | 250 | 1 |
| *Samarisporites triangulatus* | Trimerophtye or Progymnosperm spore | 250 | 1,2 |
| *Grandispora* | Zygopterid fern spore | 250 | 1,2 |
| *Auroraspora macromanifestus* | Uncertain pteridophyte spore | 250 | 1 |
| Forked axis | Rhyniopsid herb |  | 3 |
| *Leptophloeum rhombicum* | Lycopsid tree trunk | 115 | 3 |
| *Sublepidodendron mirabile* | Lycopsid tree trunk |  | 3 |
| *Sublepidodendron grabaui* | Lycopsid tree trunk |  | 3,4 |
| *Hamatophyllum verticillatum* | Equisetalean herb |  | 3 |
| *Archaeocalamites* | Equisetalean shrub |  | 3 |
| *cf. Rhacophyton ceratangium* | Zygopterid fern | 154 | here |
| *Archaeopteris macilenta* | Progymnosperm tree | 63, 115 | here |
| *Platyphyllum* | Progymnosperm seedling? |  | 3 |
| *Ningxiaphyllum trilobatum* | Pteridosperm shrub |  | 3 |
| *Planolites beverlyensis* | Worm burrow | 63 | here |
| Undetermined | Galeaspid agnathan fish |  | 5 |
| *Bothriolepis niushanensis* | Bothriolepid placoderm fish | 222 | 5 |
| *Ningxialepis spinosa* | Jiangxilepid placoderm fish | 154 | 6 |
| *Remigolepis zhongningensis* | Asterolepid placoderm fish | 167, 222, 251 | 1 |
| *Remigolepis zhongweiensis* | Asterolepid placoderm fish |  | 1 |
| *Remigolepis xiangshanensis* | Asterolepid placoderm fish |  | 1 |
| *Remigolepis major* | Asterolepid placoderm fish |  | 1 |
| *Remigolepis microcephala* | Asterolepid placoderm fish |  | 1 |
| *Remigolepis xixiaensis* | Asterolepid placoderm fish |  | 1 |
| *Sinolepis szei* | Sinolepid placoderm fish | 222 | 1 |
| *Hongyu chowi* | Elpisostegid or rhizodontid sarcopterygian fish | 167, 222 | 7 |
| *Sinostega pani* | Acanthostegid basal tetrapod | 222 | 8 |

*Note: Meter levels are from section of Fig.2 with base of Zhongning Formation at 9 m. Other fossils are from other localities. References are as follows.*

1. Pan, J., *et al. Continental Devonian System of Ningxia and its biotas*. Beijing: Geol Publ House, 237 pp. (1987).
2. Balme, B.E. Fossil in situ spores and pollen grains: an annotated catalogue. *Rev. Palaeobot. Palynol*. **87**, 81−323 (1995).
3. Zhao, X., Wu, X. & Gu, Q., Late Devonian flora from southern Ningxia. *Acta Palaeontologica Sinica*, **25**, 544−559 (1986).
4. Wang, Y. & Xu, H.H., *Sublepidodendron grabaui* comb. nov., a lycopsid from the Upper Devonian of China. *Bot. J. Linnean Soc*., **149**, 299−311 (2005).
5. Ritchie, A., *et al*. The Sinolepidae, a family of antiarchs (placoderm fishes) from the Devonian of South China and eastern Australia. *Rec. Australian Mus*. **44**, 319–370 (1992).
6. Jia, L.T., Zhu, M. & Zhao, W.J. A new antiarch fish from the Upper Devonian Zhongning Formation of Ningxia, China. *Palaeoworld*, **19**, 136−145 (2010).
7. Zhu, M. et al. A Devonian tetrapod-like fish reveals substantial parallelism in stem tetrapod evolution. *Nature Ecol. Evol*., **1**, 1470−1476 (2017).
8. Zhu, M. et al. First Devonian tetrapod from Asia. *Natur*e, **420**, 760−761 (2002).

**Table S2** Pedotypes and diagnosis for Late Devonian Zhongning Formation

| Pedotype | Dong-xiang | Diagnosis | USDA ^1^ | FAO ^2-3^ | AUSTRALIAN ^4^ |
| --- | --- | --- | --- | --- | --- |
| Fugun | fat | Red claystone with drab-haloed root traces (A) on red clay-enriched horizon (Bt) and deep (>40 cm) horizons of calcareous nodules (Bk) | Ustalf | Orthic Luvisol (Lo) | Red Brown Earth |
| Gieren | light | Orange silty claystone with drab haloed root traces (A) over orange siltstone with relict bedding (Bw). | Ochrept | Calcic Cambisol (Bk) | Brown Earth |
| Ninkian | thin | Red claystone with drab-haloed root-traces (A) over red silty shale and sandstone (C) | Fluvent | Calcaric Fluvisol (Jc) | Alluvial Soil |
| Tedzhu | button | Red claystone, with drab-haloed root traces (A) over shallow (<40 cm), horizon of calcareous nodules (Bk). | Calcid | Calcic Xerosol (Xk) | Calcareous Red Earth |
| Xulan | red | Red sandstone with red and green root traces (A) over laminated red sandstone (C) | Psamment | Calcaric Arenosol (Q) | Red sand |
| Zuzan | thick | Red, claystone (A) with gray root traces down into shallow (<50 cm) calcareous nodules in conglomerate (Bk) | Palecalcid | Calcic Xerosol (Xk) | Calcareous Red Earth |

*Note: references are*

1. Soil Survey Staff. *Keys to Soil Taxonomy*. Natural Resources Conservation Service: Washington DC, 358 pp. (2014)
2. FAO. *Soil Map of the World. Vol. VII. South Asia*. Paris: U.N.E.S.C.O., 117 pp. (1977)
3. FAO. *Soil Map of the World. Vol. VIII. North and Central Asia*. Paris: U.N.E.S.C.O., 165 pp. (1978)
4. Stace, H.C.T., Hubble, G.D., Brewer, R., Northcote, K.H., Sleeman, J.R., Mulcahy, M.J., Hallsworth, E.G. *Handbook of Australian Soils*. Rellim: Adelaide, 435 pp. (1968)

**Table S3** Interpretation of pedotypes Late Devonian Zhongning Formation

| Pedotype | Paleoclimate | Ecosystems | Parent materials | Palaeotopography | Time for formation (yrs) |
| --- | --- | --- | --- | --- | --- |
| Fugun | Subhumid with summer monsoonal rainfall | Dry woodland | Quartzofelspathic silt | Well-drained floodplain | 3,000-4,000 |
| Gieren | Semiarid | Dry woodland | Quartzofeldspathic fine sand | Alluvial levee | 1,000-2,000 |
| Ninkian | Not diagnostic for climate | Early successional vegetation | Quartzofeldspathic silt | Near-stream swale | 100-200 |
| Tedzhu | Arid with summer rainfall | Desert shrubland | Quartzofeldspathic silt | Well drained floodplain | 4,000-6,000 |
| Xulan | Not diagnostic for climate | Early successional vegetation | Quartzofeldspathic sand | Near-stream swale | 100-200 |
| Zuzan | Arid with summer monsoonal circulation | Desert shrubland | Quartzofeldspathic sand and gravel | Well drained floodplain | 10,000-20,000 |

**Table S4** Measurements of calcic palaeosols of Late Devonian Zhongning Formation

| Coordinates | Level (m) | Pedotype | Bk depth (cm) | Bk thickness (cm) | Nodule size (cm) | Burial (km) |
| --- | --- | --- | --- | --- | --- | --- |
| N37.654831 E105.995142 | 2.3 | Tedzhu | 34 | 78 | 2 | 4.278 |
| N37.654831 E105.995142 | 6.9 | Tedzhu | 36 | 63 | 1 | 4.273 |
| N37.654831 E105.995142 | 8.5 | Fugun | 53 | 100 | 3 | 4.272 |
| N37.654831 E105.995142 | 9.6 | Tedzhu | 36 | 40 | 0.9 | 4.270 |
| N37.654831 E105.995142 | 19.5 | Tedzhu | 24 | 18 | 3 | 4.261 |
| N37.654831 E105.995142 | 21 | Tedzhu | 27 | 58 | 4 | 4.259 |
| N37.654831 E105.995142 | 23.5 | Fugun | 58 | 96 | 5 | 4.257 |
| N37.654831 E105.995142 | 25 | Tedzhu | 10 | 74 | 5 | 4.255 |
| N37.654831 E105.995142 | 26 | Tedzhu | 21 | 44 | 5 | 4.254 |
| N37.654831 E105.995142 | 27 | Tedzhu | 26 | 48 | 2 | 4.253 |
| N37.654912 E105.993115 | 30.5 | Tedzhu | 36 | 85 | 5 | 4.250 |
| N37.654912 E105.993115 | 32 | Tedzhu | 25 | 36 | 6 | 4.248 |
| N37.654912 E105.993115 | 34 | Tedzhu | 24 | 76 | 7 | 4.246 |
| N37.654912 E105.993115 | 35 | Tedzhu | 36 | 54 | 2 | 4.245 |
| N37.654912 E105.993115 | 39 | Tedzhu | 24 | 23 | 3 | 4.241 |
| N37.654912 E105.993115 | 46 | Tedzhu | 25 | 45 | 2 | 4.234 |
| N37.654912 E105.993115 | 49.5 | Tedzhu | 27 | 39 | 3 | 4.231 |
| N37.654912 E105.993115 | 50.7 | Tedzhu | 13 | 42 | 3 | 4.229 |
| N37.654912 E105.993115 | 52 | Tedzhu | 24 | 44 | 1 | 4.228 |
| N37.654912 E105.993115 | 54 | Tedzhu | 29 | 49 | 1 | 4.226 |
| N37.654912 E105.993115 | 59.3 | Tedzhu | 24 | 36 | 3 | 4.221 |
| N37.654912 E105.993115 | 64 | Tedzhu | 21 | 24 | 2 | 4.216 |
| N37.654912 E105.993115 | 68.4 | Tedzhu | 27 | 36 | 1 | 4.212 |
| N37.654912 E105.993115 | 72.4 | Tedzhu | 32 | 37 | 1 | 4.208 |
| N37.654912 E105.993115 | 73.9 | Tedzhu | 24 | 33 | 1 | 4.206 |
| N37.654912 E105.993115 | 76 | Tedzhu | 25 | 36 | 1 | 4.204 |
| N37.654912 E105.993115 | 79 | Tedzhu | 26 | 44 | 2 | 4.201 |
| N37.654912 E105.993115 | 81.8 | Tedzhu | 20 | 35 | 1 | 4.198 |
| N37.654912 E105.993115 | 84 | Tedzhu | 23 | 34 | 1 | 4.196 |
| N37.654912 E105.993115 | 88.2 | Tedzhu | 30 | 45 | 1.2 | 4.192 |
| N37.654912 E105.993115 | 91 | Tedzhu | 22 | 37 | 3 | 4.189 |
| N37.654912 E105.993115 | 92.5 | Tedzhu | 24 | 36 | 1 | 4.188 |
| N37.654912 E105.993115 | 93.9 | Tedzhu | 30 | 42 | 1 | 4.186 |
| N37.654912 E105.993115 | 95.3 | Tedzhu | 27 | 38 | 1 | 4.185 |
| N37.654912 E105.993115 | 98.9 | Fugun | 54 | 50 | 1 | 4.181 |
| N37.654912 E105.993115 | 104.6 | Tedzhu | 32 | 38 | 1 | 4.175 |
| N37.654912 E105.993115 | 108.2 | Tedzhu | 15 | 23 | 1 | 4.172 |
| N37.654912 E105.993115 | 117.5 | Tedzhu | 25 | 36 | 2 | 4.163 |
| N37.654912 E105.993115 | 119.6 | Tedzhu | 22 | 31 | 0.5 | 4.160 |
| N37.654912 E105.993115 | 125.6 | Tedzhu | 26 | 40 | 1 | 4.154 |
| N37.654912 E105.993115 | 126.1 | Tedzhu | 35 | 75 | 1 | 4.154 |
| N37.654912 E105.993115 | 128.2 | Tedzhu | 26 | 40 | 1 | 4.152 |
| N37.654912 E105.993115 | 131.1 | Tedzhu | 24 | 37 | 0.8 | 4.149 |
| N37.654912 E105.993115 | 133.2 | Tedzhu | 22 | 43 | 1 | 4.147 |
| N37.654912 E105.993115 | 134.6 | Tedzhu | 19 | 24 | 0.5 | 4.145 |
| N37.654912 E105.993115 | 154.7 | Tedzhu | 26 | 36 | 0.5 | 4.125 |
| N37.654912 E105.993115 | 158.9 | Tedzhu | 36 | 52 | 1 | 4.121 |
| N37.654912 E105.993115 | 160.4 | Fugun | 54 | 68 | 0.8 | 4.120 |
| N37.654912 E105.993115 | 166.1 | Tedzhu | 22 | 42 | 1 | 4.114 |
| N37.654912 E105.993115 | 168.9 | Tedzhu | 24 | 41 | 1 | 4.111 |
| N37.654912 E105.993115 | 171.8 | Tedzhu | 30 | 41 | 0.5 | 4.108 |
| N37.654912 E105.993115 | 177.5 | Fugun | 64 | 83 | 2 | 4.103 |
| N37.654912 E105.993115 | 182 | Tedzhu | 26 | 39 | 2 | 4.098 |
| N37.654174 E105.995462 | 188 | Tedzhu | 31 | 36 | 2 | 4.092 |
| N37.654174 E105.995462 | 195 | Tedzhu | 14 | 23 | 2 | 4.085 |
| N37.654174 E105.995462 | 197.6 | Tedzhu | 28 | 33 | 2 | 4.082 |
| N37.654174 E105.995462 | 198.9 | Tedzhu | 27 | 31 | 1 | 4.081 |
| N37.654174 E105.995462 | 202.3 | Fugun | 73 | 32 | 1 | 4.078 |
| N37.654174 E105.995462 | 204.7 | Tedzhu | 33 | 48 | 2 | 4.075 |
| N37.654174 E105.995462 | 206.9 | Tedzhu | 32 | 46 | 0.5 | 4.073 |
| N37.654174 E105.995462 | 208.7 | Tedzhu | 38 | 62 | 1 | 4.071 |
| N37.654174 E105.995462 | 211.1 | Tedzhu | 35 | 63 | 2 | 4.069 |
| N37.6547364 E105.994512 | 213 | Tedzhu | 37 | 54 | 2 | 4.067 |
| N37.6547364 E105.994512 | 215.9 | Tedzhu | 33 | 52 | 2 | 4.064 |
| N37.6547364 E105.994512 | 219 | Tedzhu | 32 | 45 | 0.5 | 4.061 |
| N37.6547364 E105.994512 | 221.6 | Tedzhu | 31 | 52 | 0.5 | 4.058 |
| N37.6547364 E105.994512 | 224.7 | Fugun | 57 | 68 | 2 | 4.055 |
| N37.6547364 E105.994512 | 226.2 | Fugun | 52 | 47 | 1 | 4.054 |
| N37.6547364 E105.994512 | 227.6 | Tedzhu | 26 | 30 | 0.5 | 4.052 |
| N37.6547364 E105.994512 | 234.3 | Tedzhu | 31 | 39 | 1 | 4.046 |
| N37.6547364 E105.994512 | 236.9 | Tedzhu | 31 | 38 | 0.7 | 4.043 |
| N37.6547364 E105.994512 | 238.6 | Tedzhu | 39 | 46 | 0.8 | 4.041 |
| N37.6547364 E105.994512 | 239.7 | Tedzhu | 33 | 42 | 1 | 4.040 |
| N37.6547364 E105.994512 | 242.3 | Tedzhu | 31 | 40 | 1.2 | 4.038 |
| N37.6547364 E105.994512 | 244.8 | Tedzhu | 25 | 43 | 2 | 4.035 |
| N37.6547364 E105.994512 | 246.6 | Tedzhu | 30 | 41 | 1 | 4.033 |
| N37.6547364 E105.994512 | 247.1 | Tedzhu | 36 | 34 | 1 | 4.033 |
| N37.6547364 E105.994512 | 250.9 | Tedzhu | 30 | 45 | 0.5 | 4.029 |
| N37.6547364 E105.994512 | 253.9 | Tedzhu | 32 | 28 | 1.2 | 4.026 |
| N37.6547364 E105.994512 | 257 | Tedzhu | 28 | 41 | 1 | 4.023 |

**Table S5** Major element chemical composition (wt%) and Hg (ppm) of Late Devonian Zhongning Formation

| Sample | Level (m) | SiO2 | Al2O3 | TFe2O3 | CaO | MgO | Na2O | K2O | LOI | Total | Hg |
| --- | --- | --- | --- | --- | --- | --- | --- | --- | --- | --- | --- |
| s-1 | 1.3 | 54.06 | 13.56 | 5.16 | 5.44 | 1.41 | 0.29 | 4.12 | 15.31 | 99.35 | 0.0048 |
| s-2 | 2.1 | 40.22 | 9.28 | 3.41 | 18.03 | 1.15 | 0.21 | 2.99 | 24.00 | 99.29 | / |
| s-3 | 6.3 | 52.51 | 13.66 | 4.96 | 6.55 | 1.39 | 0.17 | 4.15 | 15.98 | 99.37 | / |
| s-4 | 6.7 | 52.7 | 13.6 | 5.10 | 7.63 | 3.15 | 0.13 | 4.08 | 12.93 | 99.32 | 0.0043 |
| s-5 | 7.1 | 52.5 | 12.1 | 3.73 | 9.02 | 1.30 | 0.20 | 3.53 | 16.99 | 99.37 | / |
| s-6 | 7.5 | 54.1 | 14.37 | 4.39 | 6.47 | 3.13 | 0.33 | 4.20 | 12.16 | 99.15 | 0.0043 |
| s-7 | 8.2 | 51.63 | 12.70 | 4.17 | 9.09 | 2.91 | 0.49 | 3.69 | 14.26 | 98.94 | / |
| s-8 | 8.4 | 47.57 | 12.70 | 4.47 | 10.45 | 2.73 | 0.79 | 3.77 | 15.99 | 98.47 | 0.0048 |
| s-9 | 9.2 | 53.95 | 14.78 | 5.07 | 6.04 | 3.18 | 0.13 | 4.40 | 11.81 | 99.36 | / |
| s-10 | 9.5 | 55.53 | 15.49 | 5.38 | 4.61 | 3.06 | 0.10 | 4.60 | 10.5 | 99.27 | 0.0039 |
| s-11 | 19.3 | 49.71 | 12.63 | 4.80 | 10.12 | 1.23 | 0.17 | 3.17 | 17.35 | 99.18 | / |
| s-12 | 19.6 | 60.63 | 15.52 | 5.90 | 2.03 | 2.9 | 0.16 | 3.79 | 8.22 | 99.15 | 0.0052 |
| s-13 | 20.4 | 18.14 | 4.41 | 1.94 | 36.84 | 0.68 | 0.17 | 0.99 | 36.31 | 99.48 | / |
| s-14 | 20.8 | 61.98 | 14.40 | 5.62 | 1.74 | 1.23 | 0.15 | 3.56 | 10.6 | 99.28 | 0.0047 |
| s-15 | 21.7 | 49.83 | 11.74 | 4.33 | 10.8 | 1.05 | 0.17 | 2.95 | 18.41 | 99.28 | / |
| s-16 | 22.3 | 43.19 | 11.54 | 4.67 | 14.95 | 1.18 | 0.12 | 2.94 | 20.76 | 99.35 | / |
| s-17 | 22.8 | 51.26 | 12.93 | 5.02 | 8.55 | 1.28 | 0.16 | 3.14 | 16.91 | 99.25 | / |
| s-18 | 23.2 | 54.04 | 12.93 | 4.72 | 7.12 | 1.29 | 0.19 | 3.16 | 15.81 | 99.26 | / |
| s-19 | 24.1 | 15.62 | 3.41 | 1.45 | 39.78 | 0.61 | 0.12 | 0.72 | 37.89 | 99.6 | 0.0047 |
| s-20 | 24.7 | 53.91 | 8.35 | 2.86 | 12.22 | 0.86 | 0.23 | 1.91 | 19.11 | 99.45 | / |
| s-21 | 26.4 | 30.41 | 6.88 | 2.58 | 26.69 | 0.92 | 0.25 | 1.47 | 30.27 | 99.47 | / |
| s-22 | 26.8 | 56.7 | 14.04 | 5.60 | 4.77 | 1.25 | 0.15 | 3.43 | 13.32 | 99.26 | 0.0038 |
| s-23 | 90.1 | 44.22 | 9.84 | 3.38 | 15.64 | 1.1 | 0.29 | 2.21 | 22.72 | 99.4 | 0.005 |
| s-24 | 90.2 | 42.9 | 11.00 | 4.36 | 15.64 | 1.17 | 0.18 | 2.71 | 21.39 | 99.35 | / |
| s-25 | 97.5 | 41.63 | 9.92 | 3.70 | 16.94 | 1.09 | 0.40 | 2.25 | 23.48 | 99.41 | / |
| s-26 | 99.2 | 44.93 | 11.27 | 4.41 | 13.64 | 1.21 | 0.3 | 2.69 | 20.93 | 99.38 | 0.0043 |
| s-27 | 118.1 | 37.59 | 8.58 | 3.29 | 20.5 | 1.17 | 0.28 | 1.97 | 25.99 | 99.37 | / |
| s-28 | 118.9 | 32.36 | 6.52 | 2.56 | 25.6 | 0.92 | 0.23 | 1.47 | 29.85 | 99.51 | / |
| s-29 | 154.2 | 54.72 | 15.21 | 6.18 | 4.69 | 2.74 | 0.39 | 4.09 | 11.28 | 99.3 | 0.0048 |
| s-30 | 155.8 | 52.25 | 13.89 | 6.54 | 6.73 | 2.75 | 0.34 | 3.73 | 13.06 | 99.29 | / |
| s-31 | 161.5 | 53.18 | 14.66 | 5.85 | 5.90 | 2.9 | 0.60 | 3.64 | 12.51 | 99.24 | / |
| s-32 | 162.8 | 54.43 | 14.69 | 5.66 | 4.95 | 2.86 | 0.57 | 3.66 | 12.44 | 99.26 | / |
| s-33 | 165.8 | 52.42 | 14.20 | 5.70 | 6.60 | 2.92 | 0.47 | 3.56 | 13.39 | 99.26 | / |
| s-34 | 167.5 | 54.4 | 13.05 | 2.95 | 7.61 | 1.98 | 0.60 | 3.03 | 15.71 | 99.33 | / |
| s-35 | 177.5 | 45.57 | 10.86 | 3.87 | 13.92 | 1.09 | 0.45 | 2.56 | 21.05 | 99.37 | / |
| s-36 | 178.3 | 45.99 | 11.20 | 4.29 | 12.89 | 1.20 | 0.44 | 2.80 | 20.52 | 99.33 | / |
| s-37 | 180.2 | 50.42 | 14.05 | 5.37 | 8.46 | 2.79 | 0.28 | 3.63 | 14.28 | 99.28 | / |
| s-38 | 181.5 | 50.69 | 14.24 | 6.06 | 7.67 | 2.84 | 0.25 | 3.83 | 13.7 | 99.28 | / |
| s-41 | 199.5 | 53.05 | 14.53 | 5.86 | 6.32 | 2.86 | 0.21 | 4.02 | 12.43 | 99.28 | / |
| s-42 | 200.1 | 39.12 | 8.89 | 3.73 | 19.62 | 1.25 | 0.27 | 2.37 | 23.92 | 99.17 | / |
| s-39 | 201.8 | 52.57 | 9.74 | 2.25 | 11.39 | 1.08 | 0.59 | 1.89 | 19.86 | 99.37 | 0.0062 |
| s-43 | 203.3 | 41.24 | 6.39 | 2.23 | 20.25 | 0.71 | 0.68 | 1.22 | 26.71 | 99.43 | / |
| s-40 | 203.4 | 47.07 | 10.35 | 4.34 | 12.52 | 1.11 | 0.42 | 2.3 | 21.28 | 99.39 | / |
| s-44 | 204.1 | 45.74 | 10.24 | 4.28 | 12.88 | 1.20 | 0.47 | 2.59 | 21.64 | 99.04 | 0.0073 |
| s-45 | 217.3 | 46.6 | 11.90 | 5.12 | 11.87 | 3.24 | 0.31 | 3.34 | 16.93 | 99.31 | / |
| s-46 | 217.9 | 44.63 | 9.82 | 3.62 | 13.95 | 1.27 | 0.44 | 2.59 | 23.06 | 99.38 | / |
| s-47 | 224.3 | 48.58 | 13.05 | 5.68 | 9.31 | 3.51 | 0.35 | 3.73 | 15.09 | 99.3 | / |
| s-48 | 225.8 | 46.59 | 10.49 | 4.38 | 12.23 | 1.55 | 0.39 | 2.90 | 20.83 | 99.36 | / |
| s-49 | 226.8 | 47.1 | 10.65 | 4.56 | 12.37 | 2.97 | 0.49 | 2.70 | 18.46 | 99.3 | / |
| s-50 | 227.5 | 47.05 | 11.13 | 4.00 | 12.24 | 3.46 | 0.43 | 2.74 | 18.27 | 99.32 | 0.0046 |

**Table S6** Trace element chemical composition (As-Ni, ppm) Late Devonian Zhongning Formation

| Sam-ple | Level (m) | As | Ba | Bi | Br | Ce | Cl | Co | Cr | Cu | F | Ga | Hf | La | Mn | Mo | Nb | Ni |
| --- | --- | --- | --- | --- | --- | --- | --- | --- | --- | --- | --- | --- | --- | --- | --- | --- | --- | --- |
| s-1 | 1.3 | 4.6 | 685.3 | / | 0.7 | 52.5 | 8.9 | 17.0 | 66.8 | 11.1 | 907.5 | 16.6 | 4.8 | 27.0 | 445.0 | 0.9 | 12.4 | 33.9 |
| s-2 | 2.1 | 2.6 | 435.4 | / | 1.2 | 0.5 | 675 | 11.9 | 56.9 | 22.4 | 719.8 | 13.1 | 3.8 | 10.2 | 893.6 | 1.4 | 10.2 | 35.8 |
| s-3 | 6.3 | 7.9 | 377.6 | / | 1.0 | 45.8 | / | 15.4 | 69.7 | 7.0 | 875.1 | 17.0 | 4.8 | 25.0 | 518.5 | 1.1 | 11.5 | 36.0 |
| s-4 | 6.7 | 8.2 | 403.1 | / | 0.3 | 34.8 | 11 | 16.2 | 66.9 | 8.9 | 877.9 | 17.8 | 4.8 | 22.8 | 674.4 | 1.1 | 12.8 | 36.0 |
| s-5 | 7.1 | 5.2 | 386.3 | / | 0.3 | 32.2 | 25.9 | 13.4 | 59.4 | 8.9 | 716.8 | 14.1 | 5.3 | 19.5 | 662.5 | 1.1 | 12.2 | 29.9 |
| s-6 | 7.5 | 6.2 | 430.6 | / | 0.7 | 46.2 | 1190.8 | 13.5 | 61.1 | 9.1 | 894.7 | 17.0 | 5.9 | 21.9 | 718.0 | 1.0 | 14.2 | 26.8 |
| s-7 | 8.2 | 4.8 | 339 | / | 1.0 | 43.2 | 3972 | 12.8 | 61.3 | 9.5 | 736.9 | 15.1 | 5.4 | 20.7 | 621.9 | 1.4 | 12.8 | 29.7 |
| s-8 | 8.4 | 6.2 | 458.5 | / | 0.3 | / | 8300.3 | 14.2 | 67.5 | 9.4 | 735.7 | 15.3 | 4.7 | / | 892.4 | 1.6 | 10.9 | 31.0 |
| s-9 | 9.2 | 6.3 | 381.8 | / | 0.9 | 41.7 | 289.5 | 16.2 | 64.2 | 11.7 | 890.3 | 17.0 | 5.0 | 21.2 | 393.1 | 0.9 | 11.5 | 31.5 |
| s-10 | 9.5 | 8.6 | 581.7 | / | 0.8 | 45.2 | 168.2 | 19.3 | 67.6 | 54.1 | 955.8 | 18.0 | 4.8 | 26.3 | 1004.1 | 2.8 | 11.9 | 34.8 |
| s-11 | 19.3 | 8.0 | 502.7 | / | 0.7 | 25.7 | 534.1 | 18.3 | 73.8 | 18.5 | 776.9 | 16.8 | 6.5 | 24.2 | 301.7 | 0.8 | 14.3 | 35.6 |
| s-12 | 19.6 | 10.2 | 663.4 | / | 0.3 | 85.7 | 224.9 | 24.1 | 78.8 | 8.5 | 936.7 | 20.0 | 8.0 | 42.0 | 153.5 | 1.3 | 17.1 | 36.8 |
| s-13 | 20.4 | 4.2 | 191.7 | / | 1.2 | / | 543.8 | 6.7 | 32.4 | 19.7 | 418 | 6.6 | 3.3 | 2.7 | 725.2 | 0.7 | 8.1 | 21.6 |
| s-14 | 20.8 | 12.4 | 686 | / | 0.9 | 74 | 1.7 | 22.3 | 76.7 | 10.4 | 943.8 | 17.8 | 7.1 | 34.6 | 138.2 | 1.5 | 16.3 | 32.0 |
| s-15 | 21.7 | 9.9 | 524.8 | / | 0.4 | 55.6 | 10.5 | 15.6 | 68.4 | 11.8 | 704.9 | 14.4 | 7.5 | 29.3 | 310 | 0.7 | 14.1 | 28.1 |
| s-16 | 22.3 | 8.4 | 459.7 | 0.1 | 1.0 | 26.9 | 12.3 | 18.8 | 80 | 14.4 | 756.7 | 15.8 | 5.0 | 14.0 | 346.4 | 1.4 | 13.6 | 39.0 |
| s-17 | 22.8 | 14.2 | 602.8 | / | 0.7 | 49.4 | 2.2 | 16.7 | 73.7 | 10.7 | 746.1 | 15.9 | 6.6 | 26.0 | 319.8 | 0.5 | 15.0 | 36.6 |
| s-18 | 23.2 | 17.7 | 603.3 | / | 0.2 | 49.4 | 74.3 | 17.0 | 66.3 | 6.9 | 760.1 | 16.6 | 6.1 | 29.4 | 333.1 | 1.4 | 15.2 | 33.8 |
| s-19 | 24.1 | 2.6 | 186.7 | / | 0.8 | / | 98.9 | 6.2 | 25.6 | 33.5 | 326.9 | 4.7 | 3.2 | 0.1 | 686.7 | 1.1 | 5.9 | 17.3 |
| s-20 | 24.7 | 5.5 | 459.7 | / | / | 33.7 | 4.1 | 14.4 | 41.9 | 16.4 | 500 | 9.7 | 6.4 | 19.0 | 514.7 | 1.0 | 10.4 | 21.6 |
| s-21 | 26.4 | 5.2 | 318.7 | / | 0.2 | / | 17.6 | 9.2 | 51.1 | 18.9 | 410.2 | 9.3 | 4.0 | 6.3 | 722.2 | 0.1 | 8.8 | 26.7 |
| s-22 | 26.8 | 9.1 | 803.6 | / | 1.1 | 58.6 | 38.5 | 19.7 | 74.1 | 12.1 | 835.5 | 17.4 | 6.7 | 31.7 | 191.7 | 0.6 | 15.0 | 34.7 |
| s-23 | 90.1 | 5.2 | 423.1 | / | 1.1 | 24.4 | 14 | 13.7 | 62.1 | 14.3 | 576.3 | 13.3 | 6.6 | 18.0 | 475.2 | 1.0 | 11.6 | 28.0 |
| s-24 | 90.2 | 9.6 | 477.8 | / | 0.3 | 19.0 | 7.4 | 14.8 | 67.7 | 14.5 | 701.4 | 14.1 | 5.5 | 15.5 | 469.6 | 1.0 | 12.7 | 36.2 |
| s-25 | 97.5 | 6.4 | 363.7 | / | 0.7 | 20.7 | 2.4 | 13.3 | 59.2 | 12.0 | 572.8 | 13.4 | 4.9 | 14.0 | 539.9 | 1.7 | 11.2 | 29.8 |
| s-26 | 99.2 | 8.3 | 434.2 | / | 0.8 | 19.1 | 2.8 | 15.2 | 59.3 | 10.3 | 613.4 | 15.8 | 5.1 | 19.2 | 444.4 | 1.2 | 13.1 | 34.1 |
| s-27 | 118.1 | 4.2 | 408.4 | / | 0.9 | 9.9 | 249.1 | 11.3 | 61.8 | 11.5 | 532.3 | 12.0 | 5.1 | 9.5 | 675.9 | 1.3 | 11.2 | 28.5 |
| s-28 | 118.9 | 3.6 | 377.8 | / | 0.7 | 7.2 | 108.7 | 10.9 | 47 | 14.4 | 458.1 | 10.1 | 4.6 | 6.9 | 595.0 | 1.7 | 9.1 | 22.8 |
| s-29 | 154.2 | 9.0 | 390.2 | / | / | 47.2 | 3.1 | 17.9 | 70.2 | 13.0 | 1089.2 | 22.9 | 6.9 | 28.4 | 268.7 | 1.3 | 17.3 | 34.3 |
| s-30 | 155.8 | 24.6 | 375.8 | / | 1.4 | 36.0 | / | 19.4 | 75.2 | 10.4 | 1060.4 | 20.9 | 6.3 | 22.0 | 306 | 1.1 | 15.8 | 34.0 |
| s-31 | 161.5 | 6.3 | 343.8 | / | 0.4 | 58.1 | 44.2 | 17.6 | 79.9 | 7.0 | 840.9 | 21.4 | 5.8 | 31.0 | 424.3 | 1.5 | 16.6 | 39.7 |
| s-32 | 162.8 | 6.9 | 381.4 | / | 0.8 | 64.3 | 35.1 | 17.6 | 71.8 | 6.2 | 929.5 | 20.7 | 6.9 | 34.9 | 359.8 | 0.7 | 16.0 | 36.8 |
| s-33 | 165.8 | 11.2 | 397.3 | / | 1.2 | 55.4 | 8.9 | 18.7 | 68.7 | 8.9 | 871 | 18.9 | 5.8 | 27.8 | 672.4 | 2.3 | 14.2 | 37.7 |
| s-34 | 167.5 | 3.0 | 275 | / | 1.0 | 68.8 | 13.2 | 13.2 | 67.5 | 8.8 | 750 | 16.9 | 6.7 | 37.7 | 399.8 | 1.1 | 14.0 | 29.5 |
| s-35 | 177.5 | 5.1 | 246.4 | / | / | 43.7 | 4.5 | 12.8 | 57.5 | 16.4 | 664.9 | 13.5 | 5.4 | 24.4 | 586.9 | 1.1 | 12.6 | 30.8 |
| s-36 | 178.3 | 4.5 | 427.1 | / | 1.2 | 34.9 | 18.1 | 14.4 | 66.1 | 33.6 | 651.8 | 14.8 | 5.1 | 18.4 | 557.5 | 1.2 | 13.2 | 33.3 |
| s-37 | 180.2 | 6.0 | 395.6 | 0.9 | 0.9 | 53.1 | / | 16.6 | 73.7 | 17.3 | 964.8 | 18.4 | 5.8 | 29.4 | 378.5 | 0.9 | 14.5 | 35.5 |
| s-38 | 181.5 | 6.6 | 375.4 | / | 0.7 | 50.0 | 5.5 | 18.2 | 74.3 | 21.9 | 967.2 | 20.7 | 5.6 | 27.9 | 376.3 | 1.5 | 15.2 | 38.2 |
| s-41 | 199.5 | 6.3 | 348 | / | 0.5 | 35.1 | 13.5 | 16.5 | 75.1 | 20.2 | 1013.1 | 19.9 | 5.4 | 21.8 | 246.5 | 1.0 | 15.0 | 39.0 |
| s-42 | 200.1 | 11.4 | 427 | / | 1.1 | 24.7 | 1367.6 | 11.4 | 56.8 | 21.1 | 626.1 | 12.3 | 5.7 | 12.1 | 611.4 | 2.8 | 11.2 | 28.4 |
| s-39 | 201.8 | 7.0 | 169.7 | / | 0.9 | 63.2 | 75 | 10.5 | 51.2 | 30.0 | 565.9 | 12 | 8.1 | 32.0 | 273.5 | 1.4 | 11.7 | 23.6 |
| s-43 | 203.3 | 2.9 | 139.5 | 0.7 | 1.0 | 37.3 | 583.7 | 9.9 | 33.6 | 16.4 | 476.7 | 6.7 | 4.9 | 20.0 | 593.3 | 1.7 | 9.7 | 17.3 |
| s-40 | 203.4 | 4.8 | 196.6 | / | 0.1 | 43.3 | 16.1 | 13.4 | 60.9 | 47.7 | 648.3 | 14.8 | 5.6 | 25.6 | 253.4 | 0.6 | 13.3 | 28.3 |
| s-44 | 204.1 | 6.8 | 193.7 | / | 0.8 | 37.8 | 189.1 | 13.7 | 64 | 16.7 | 713.7 | 15.7 | 4.7 | 22.3 | 638.1 | 1.7 | 12.3 | 31.4 |
| s-45 | 217.3 | 5.9 | 343 | / | 0.8 | 30.6 | 11.2 | 15.0 | 65.3 | 8.5 | 828.9 | 17.7 | 4.2 | 22.0 | 514.8 | 0.5 | 12.4 | 39.7 |
| s-46 | 217.9 | 5.5 | 228.9 | / | 0.7 | 20.3 | 19.6 | 12.8 | 57.9 | 9.4 | 802.2 | 13.4 | 5.2 | 12.9 | 460 | 0.7 | 12.1 | 26.4 |
| s-47 | 224.3 | 6.6 | 275.6 | / | 0.7 | 51.0 | 7.6 | 15.9 | 71.8 | 8.9 | 911 | 17.1 | 4.9 | 26.5 | 440.9 | 0.6 | 13.6 | 36.2 |
| s-48 | 225.8 | 6.5 | 341.3 | 0 | 0.5 | 45.9 | 13.6 | 14.8 | 60.3 | 9.6 | 729.1 | 14.0 | 4.6 | 24.6 | 467.3 | 0.7 | 12.3 | 28.7 |
| s-49 | 226.8 | 5.6 | 199.5 | / | 0.9 | 63.2 | 12.3 | 13.4 | 62.6 | 67 | 779.7 | 15.7 | 5.1 | 34.2 | 595.4 | 1.8 | 12.8 | 29.8 |
| s-50 | 227.5 | 3.3 | 171.8 | / | 1.5 | 65.8 | 6.5 | 14.4 | 61.1 | 70.4 | 683.7 | 14.7 | 5.2 | 33.4 | 544.4 | 0.8 | 12.8 | 32.2 |

**Table S7** Trace element chemical composition (P-Zr, ppm) of Late Devonian Zhongning Formation

| Sam-ple | Level (m) | P | Pb | Rb | S | Sb | Sc | Sn | Sr | Ta | Ti | Th | U | V | W | Y | Zn | Zr |
| --- | --- | --- | --- | --- | --- | --- | --- | --- | --- | --- | --- | --- | --- | --- | --- | --- | --- | --- |
| s-1 | 1.3 | 469.9 | 21 | 152.3 | 116.4 | / | 8.9 | 2.2 | 99.5 | 1.7 | 3151.7 | 13.5 | 3.1 | 80.0 | 7.6 | 19.3 | 74.4 | 129.3 |
| s-2 | 2.1 | 389.3 | 14.9 | 109.8 | 452.3 | 4 | 7.7 | 0.9 | 172.7 | 1.8 | 2686.6 | 13.8 | 1.7 | 72.7 | 5.5 | 17.1 | 58.9 | 98.8 |
| s-3 | 6.3 | 406.6 | 22.2 | 153.4 | 117.3 | / | 8.2 | 2.6 | 99.5 | 1.7 | 3076.7 | 12.0 | 3.1 | 79.6 | 6.9 | 18.4 | 74.3 | 135.2 |
| s-4 | 6.7 | 429.1 | 23.9 | 154.0 | 147.4 | 0.8 | 8.4 | 8.0 | 117.9 | 1.8 | 3352.0 | 15.0 | 2.5 | 80.8 | 7.4 | 20.1 | 78 | 138 |
| s-5 | 7.1 | 432.4 | 15.3 | 130.1 | 396.9 | 0.3 | 9.6 | 0.9 | 130.3 | 1.6 | 2832.5 | 12.6 | 2.7 | 70.1 | 6.8 | 21.0 | 64.9 | 161.3 |
| s-6 | 7.5 | 405.3 | 22.8 | 154.8 | 908.7 | / | 9.1 | 7.6 | 112.5 | 1.5 | 3076.4 | 13.6 | 2.9 | 75.8 | 7.2 | 24.3 | 69.7 | 188.7 |
| s-7 | 8.2 | 415.5 | 18.3 | 139.7 | 446.1 | 0.4 | 7.7 | 3.0 | 136.9 | 1.6 | 3115.9 | 11.9 | 1.3 | 70.0 | 6.4 | 22.4 | 67.2 | 169.1 |
| s-8 | 8.4 | 435.4 | 19.8 | 140.3 | 686.6 | 2.0 | 7.1 | 4.7 | 126.8 | 1.7 | 3038.7 | 13.1 | 3.8 | 74.6 | 6.3 | 21.7 | 64.4 | 129.9 |
| s-9 | 9.2 | 410.6 | 19.8 | 154.3 | 308 | 2.9 | 8.3 | 2.5 | 76.2 | 1.6 | 3016.8 | 12.9 | 2.4 | 68.4 | 8 | 18.9 | 70.6 | 141.2 |
| s-10 | 9.5 | 452.1 | 32.3 | 160.4 | 124.7 | / | 8.3 | 2.7 | 67.8 | 1.6 | 3140.9 | 12.9 | 2.6 | 78.7 | 7.9 | 19.2 | 68.9 | 139.3 |
| s-11 | 19.3 | 526.4 | 15.8 | 139.0 | 874.9 | 1.4 | 8 | 4.0 | 77.6 | 1.7 | 3888.7 | 16.4 | 2.8 | 89.4 | 8.9 | 23.6 | 75.2 | 193.7 |
| s-12 | 19.6 | 512.7 | 18.5 | 168.5 | 420.9 | 1.5 | 9.5 | 4.6 | 58.6 | 1.8 | 4488.5 | 18.6 | 3.2 | 87.6 | 11.3 | 24.5 | 77.0 | 280.6 |
| s-13 | 20.4 | 313.6 | 11.2 | 42.8 | 687.7 | 0.2 | 1.4 | / | 195.8 | 1.6 | 1788.5 | 8.4 | 0.4 | 46.0 | 2.1 | 12.1 | 34.0 | 86.3 |
| s-14 | 20.8 | 367.4 | 23.5 | 161.2 | 120.6 | 1.7 | 10.2 | 5.3 | 48.4 | 1.8 | 4069.4 | 17.0 | 3.8 | 86.1 | 9.8 | 22.4 | 73.4 | 229.7 |
| s-15 | 21.7 | 660 | 20.2 | 126.8 | 108.4 | 2.4 | 9.9 | 2.1 | 70.3 | 1.6 | 3852.9 | 15.1 | 3.2 | 83.0 | 7.2 | 20.5 | 59.2 | 254.1 |
| s-16 | 22.3 | 429.3 | 17.6 | 135.3 | 115 | 4.6 | 8.0 | 1.8 | 65.5 | 1.9 | 3597.6 | 12.6 | 1.7 | 95.2 | 9.5 | 25.0 | 79.7 | 151.6 |
| s-17 | 22.8 | 607.7 | 20.6 | 140.8 | 115.3 | 3.8 | 7.4 | 2.4 | 69.5 | 1.8 | 4105.2 | 18.6 | 3.9 | 86.3 | 7.4 | 26.7 | 79.1 | 215.7 |
| s-18 | 23.2 | 572.1 | 19.5 | 139.7 | 357.4 | 1.2 | 9.9 | 5.2 | 70.9 | 1.6 | 3837.0 | 16.5 | 2.6 | 82.0 | 8.4 | 26.0 | 75.9 | 205.9 |
| s-19 | 24.1 | 255.6 | 13.3 | 31.7 | 530.5 | / | 1.3 | / | 196.3 | 1.6 | 1414.7 | 7.9 | 1.5 | 30.8 | 1.7 | 13.4 | 33.0 | 80.4 |
| s-20 | 24.7 | 614.3 | 16.3 | 79.0 | 81.6 | / | 2.6 | 1.6 | 81.2 | 1.4 | 2579.5 | 10.6 | 2.2 | 55.8 | 8.2 | 17.7 | 42.9 | 205.6 |
| s-21 | 26.4 | 374.5 | 13.1 | 63.8 | 298.4 | 1 | 2.1 | 3.5 | 141.2 | 1.7 | 2409 | 12.2 | 0.8 | 55.6 | 4.5 | 18.0 | 51.6 | 111.7 |
| s-22 | 26.8 | 516.4 | 19.6 | 157.3 | 169.3 | / | 11.6 | 2.3 | 56.7 | 1.7 | 3904.8 | 16.9 | 1.3 | 86.8 | 8.9 | 25.6 | 83.8 | 217.4 |
| s-23 | 90.1 | 456.6 | 14.7 | 100.8 | 119 | 0.6 | 7.6 | 1.8 | 109.5 | 1.5 | 3276.4 | 15.1 | 3.1 | 69.6 | 7.0 | 21.4 | 60.4 | 207.3 |
| s-24 | 90.2 | 489.9 | 22.6 | 125.8 | 91.8 | 3.8 | 4.9 | 1.1 | 122.6 | 1.8 | 3490.6 | 15.4 | 0.8 | 74.3 | 6.9 | 21.6 | 71.3 | 168.8 |
| s-25 | 97.5 | 444.9 | 21.9 | 102.2 | 101.1 | 0.2 | 3.4 | 3.0 | 126.5 | 1.7 | 3064.6 | 12.2 | 1.3 | 67.2 | 7.1 | 21.1 | 65.2 | 158.9 |
| s-26 | 99.2 | 516.5 | 20.1 | 124.5 | 70.2 | / | 5.8 | 1.4 | 106.4 | 1.8 | 3326.9 | 12.1 | 1.8 | 74.3 | 7.1 | 21.8 | 74.9 | 148.8 |
| s-27 | 118.1 | 371.9 | 14.5 | 89.4 | 174.8 | 1.8 | 7.8 | 3.0 | 166.4 | 1.6 | 3098.7 | 12.9 | 0.8 | 76.2 | 5.4 | 19.9 | 59.3 | 152.8 |
| s-28 | 118.9 | 323 | 14.5 | 65.3 | 152.9 | / | 5.9 | / | 159.7 | 1.6 | 2345.1 | 9.3 | 1.4 | 59.8 | 5.2 | 18.6 | 46.9 | 132.4 |
| s-29 | 154.2 | 397.5 | 31.3 | 196.3 | 45.7 | 2.2 | 10.8 | 7.8 | 65.8 | 1.9 | 3800.6 | 18.9 | 4.9 | 79.5 | 7.9 | 30.4 | 88.3 | 222.7 |
| s-30 | 155.8 | 480.1 | 34.1 | 177.5 | 67.2 | 0.3 | 10.0 | 2.0 | 81.0 | 1.8 | 3747.5 | 17.3 | 4.5 | 97.3 | 8.0 | 30.1 | 88.6 | 194.1 |
| s-31 | 161.5 | 518 | 26.3 | 175.3 | 189.9 | 1.5 | 8.9 | 8.5 | 89.3 | 1.9 | 4176.7 | 20.8 | 1.9 | 80.1 | 8.4 | 29.4 | 96.4 | 182.0 |
| s-32 | 162.8 | 628.2 | 27.1 | 168.7 | 177.5 | 2 | 11.4 | 2.7 | 80.2 | 1.7 | 3939.6 | 20.2 | 3.2 | 79.5 | 8.7 | 29.3 | 87.6 | 213.2 |
| s-33 | 165.8 | 533.5 | 41.4 | 163.9 | 109.5 | 0.7 | 6.9 | 0.1 | 81.3 | 1.7 | 3800.8 | 16.9 | 4.3 | 88.9 | 7.8 | 25.6 | 81.5 | 180.0 |
| s-34 | 167.5 | 567.8 | 12.0 | 135.4 | 153.0 | / | 9.0 | 4.3 | 88.5 | 1.3 | 3644.9 | 16.6 | 1.8 | 107.9 | 9.2 | 23.1 | 78.2 | 223.9 |
| s-35 | 177.5 | 498 | 23.3 | 115.4 | 164.0 | 1.7 | 5.7 | 7.7 | 138.7 | 1.6 | 3430.9 | 15.7 | 2.1 | 72.2 | 6.2 | 24.8 | 67.1 | 162.7 |
| s-36 | 178.3 | 474.4 | 21.0 | 127.4 | 287.7 | 2.4 | 7.5 | 2.9 | 150.8 | 1.7 | 3486.7 | 15.5 | 3.1 | 86.9 | 6.6 | 23.3 | 74.4 | 171.1 |
| s-37 | 180.2 | 520.9 | 20.3 | 168.5 | 87.7 | 1.4 | 6.3 | 1.2 | 103.7 | 1.9 | 3895.4 | 18.2 | 1.9 | 87.4 | 8.5 | 25.7 | 91.5 | 176.0 |
| s-38 | 181.5 | 522.1 | 20.1 | 178.1 | 82.7 | 3.1 | 11.5 | 3.8 | 88.2 | 1.8 | 3879.4 | 18.9 | 2.2 | 91.3 | 8.1 | 27.8 | 91.5 | 171.3 |
| s-41 | 199.5 | 596 | 24.6 | 185.7 | 81.0 | 4.3 | 8.9 | 6.9 | 74.5 | 1.8 | 4004.8 | 17.9 | 3.2 | 101.0 | 7.3 | 25.6 | 89.3 | 165.9 |
| s-42 | 200.1 | 475.3 | 23.8 | 104.8 | 814.5 | / | 5.8 | / | 112.7 | 1.7 | 3123.0 | 13.4 | 2.6 | 103.0 | 6.9 | 23.2 | 75.9 | 173.5 |
| s-39 | 201.8 | 664.1 | 11.9 | 82.5 | 543.6 | 1.5 | 6.0 | 4.4 | 97 | 1.4 | 3112.8 | 11.9 | 1.5 | 68.5 | 8.5 | 24.8 | 65.6 | 277.5 |
| s-43 | 203.3 | 452 | 19.6 | 50.3 | 212.3 | / | 4.0 | / | 192 | 1.4 | 2552.7 | 9.0 | 2.2 | 51.0 | 4.0 | 18.3 | 34.3 | 151.8 |
| s-40 | 203.4 | 538 | 24.5 | 103 | 356.1 | 4.2 | 7.0 | 3.0 | 108.9 | 1.8 | 3378.6 | 13.9 | 1.2 | 73.1 | 6.9 | 23.0 | 72.6 | 176.5 |
| s-44 | 204.1 | 425.9 | 26.7 | 115 | 3046.2 | / | 6.9 | 1.1 | 273.1 | 1.7 | 3433.4 | 11.6 | 0.9 | 85.3 | 6.3 | 21.0 | 68.7 | 151.0 |
| s-45 | 217.3 | 476.3 | 24.5 | 149.2 | 144.5 | / | 4.5 | 7.0 | 173.5 | 1.8 | 3729.3 | 16.3 | 1.8 | 81.6 | 7.8 | 23.6 | 89.8 | 133.4 |
| s-46 | 217.9 | 506.1 | 16.0 | 109.2 | 123.2 | / | 4.7 | 1.5 | 198 | 1.5 | 3156.9 | 14.8 | 1.9 | 65.8 | 7.0 | 22.2 | 67.0 | 176.1 |
| s-47 | 224.3 | 615.3 | 29.0 | 167.5 | 97.8 | / | 6.5 | 5.2 | 164.4 | 1.8 | 3770.7 | 16.7 | 2.4 | 81.7 | 7.6 | 24.6 | 91.8 | 136.5 |
| s-48 | 225.8 | 571.6 | 23.0 | 122.5 | 159.8 | 0.2 | 5.9 | / | 193.2 | 1.6 | 3270.6 | 11.7 | 2.0 | 79.1 | 7.7 | 21.7 | 71.4 | 149.3 |
| s-49 | 226.8 | 610.8 | 26.0 | 116.5 | 165.1 | / | 3.7 | / | 187.6 | 1.6 | 3629.3 | 14.0 | 2.5 | 81.7 | 7.3 | 22.6 | 79.5 | 150.7 |
| s-50 | 227.5 | 581.5 | 16.0 | 119.1 | 187.5 | 0.8 | 5.5 | 4.2 | 191.3 | 1.7 | 3607.7 | 15.0 | 2.4 | 89.7 | 8.9 | 23.2 | 85.0 | 171.4 |
